# Host and Viral Factors in HIV-Mediated Bystander Apoptosis

**DOI:** 10.3390/v9080237

**Published:** 2017-08-22

**Authors:** Himanshu Garg, Anjali Joshi

**Affiliations:** Center of Emphasis in Infectious Diseases, Department of Biomedical Sciences, Texas Tech University Health Sciences Center, 5001 El Paso Dr., El Paso, TX 79905, USA

**Keywords:** HIV, AIDS, apoptosis, bystander, Env, CCR5, immune activation, fusion, hemifusion, gp41

## Abstract

Human immunodeficiency virus (HIV) infections lead to a progressive loss of CD4 T cells primarily via the process of apoptosis. With a limited number of infected cells and vastly disproportionate apoptosis in HIV infected patients, it is believed that apoptosis of uninfected bystander cells plays a significant role in this process. Disease progression in HIV infected individuals is highly variable suggesting that both host and viral factors may influence HIV mediated apoptosis. Amongst the viral factors, the role of Envelope (Env) glycoprotein in bystander apoptosis is well documented. Recent evidence on the variability in apoptosis induction by primary patient derived Envs underscores the role of Env glycoprotein in HIV disease. Amongst the host factors, the role of C-C Chemokine Receptor type 5 (CCR5), a coreceptor for HIV Env, is also becoming increasingly evident. Polymorphisms in the *CCR5* gene and promoter affect CCR5 cell surface expression and correlate with both apoptosis and CD4 loss. Finally, chronic immune activation in HIV infections induces multiple defects in the immune system and has recently been shown to accelerate HIV Env mediated CD4 apoptosis. Consequently, those factors that affect CCR5 expression and/or immune activation in turn indirectly regulate HIV mediated apoptosis making this phenomenon both complex and multifactorial. This review explores the complex role of various host and viral factors in determining HIV mediated bystander apoptosis.

## 1. Introduction

Human immunodeficiency virus (HIV) infection in humans leads to a progressive loss of CD4 T cells culminating in immunodeficiency. While HIV is known to selectively infect CD4 cells, the mechanism of CD4 T cell loss is more complex than virus infection alone. Mounting evidence suggests that both and host and viral factors play a significant role in determining CD4 T cell loss. Recent studies have started to unravel this complex interplay and a better picture is emerging, which can to a great extent explain the selectivity of CD4 loss as well as the variations in disease progression. Fundamental to HIV pathogenesis is the phenomenon of apoptosis that is believed to be a major pathway in CD4 loss. This review looks at the various host and viral factors that play a role in regulating CD4 apoptosis in HIV infections.

**Apoptosis in HIV infections:** The phenomenon of apoptosis in HIV infections has been observed from the earliest days of HIV research [1,2,3]. Support for a role of apoptosis in CD4 T cell loss in HIV infections also comes from simian immunodeficiency virus (SIV) infections in non-human primates. Pathogenic SIV infection in macaques is characterized by increased apoptosis, which is remarkably absent in non-pathogenic SIV infection in natural host [4,5,6,7]. In the SIV/HIV chimeric SHIV virus infection in macaques, a correlation between apoptosis and CD4 loss is also evident [8,9]. Multiple studies have reported increased apoptosis in peripheral blood mononuclear cells (PBMCs) from HIV infected individuals that correlates with CD4 decline [10,11,12]. Although the involvement of apoptosis in CD4 decline in HIV infections is widely accepted, the mechanism of apoptosis induction remains debated.

**Role of virus replication in apoptosis:** Immunopathogenic features of HIV infection, including CD4 loss in some cases correlate poorly with viremia [13,14,15]. Furthermore, high levels of viremia in natural SIV infections fails to induce CD4 apoptosis and subsequent CD4 loss [4]. These observations argue against a role of viremia in HIV mediated apoptosis. On the other hand, a role of viremia in CD4 apoptosis is supported by the observation that suppression of virus replication with highly active antiretroviral therapy (HAART) can reverse both CD4 loss as well as T cells apoptosis in the peripheral blood [16,17,18]. Similarly, untreated HIV infections have been shown to be associated with high levels of apoptosis [11]. Recent evidence from our lab demonstrates that a number of immunopathological features of HIV infection including CD4 apoptosis correlates with viremia [10]. The success of HAART in delaying acquired immunodeficiency syndrome (AIDS) onset and increasing life expectancy in HIV infected individuals is probably the strongest evidence for a role of viremia in HIV pathology including CD4 T cell decline [19,20]. At the same time, this process is complex and cases where viremia fails to mediate CD4 apoptosis/decline; other host and viral factors are likely involved.

**Bystander Apoptosis:** The number of HIV infected cells in patients is relatively low and cannot solely account for the loss of CD4 cells in vivo. Hence, it is believed that the loss of CD4 cells during HIV infection is due to the process of bystander apoptosis induction [3,21,22]. Early studies by Finkel et al. [23] demonstrated that the majority of cells undergoing apoptosis during HIV infection are not infected but in close proximity to infected cells. A role of direct infection in loss of CD4 cells is also refuted by SIV infection in natural host where high levels of infection and viremia do not result in AIDS development [24]. Hence, bystander apoptosis is believed to be one of the major causes of CD4 loss leading to AIDS [3,22,25]. In vitro infection of cell lines and PBMCs with HIV-1 also shows that apoptosis is largely restricted to uninfected bystander cells [26,27,28]. Furthermore, in pathogenic SIV models [5,6] and HIV infection in humanized mouse models [26,29], apoptosis in bystander cells has been observed.

## 2. Viral Factors

### 2.1. Env Glycoprotein

The role of Envelope (Env) glycoprotein in mediating bystander apoptosis has been extensively studied [21,30,31]. The involvement of Env glycoprotein in this process is supported by three major observations. Firstly, as cell death in HIV infection outnumbers the infected cell population, a role of bystander T cell apoptosis in progression to AIDS has been proposed. Secondly, as the depletion of immune cells is largely restricted to CD4 T cells and as the Env glycoprotein binds directly to CD4, it likely plays a role in CD4 T cell death. Finally, Env glycoprotein is expressed on the surface of infected cells and has been shown to interact with bystander cells expressing CD4 and a coreceptor CXCR4/CCR5 to mediate apoptosis [32,33,34,35,36]. Thus, the Env glycoprotein is believed to be a major player in HIV induced cell death.

The structural features of HIV Env are fundamental to its ability to mediate bystander apoptosis. The Env glycoprotein of HIV is arranged on the surface of the virus and virus-infected cells as a hetero-trimer. Each monomer is composed of a receptor-binding gp120 unit and a gp41 transmembrane unit that mediates fusion of viral and cellular membrane [37]. The sequence of events that lead to fusion catalyzed by HIV-1 Env are initiated by the binding of gp120 subunit to CD4 and a coreceptor, either CXCR4 or CCR5, on CD4 cells. This binding of HIV gp120 to CD4 triggers several conformational changes in gp120 that result in exposure of coreceptor binding site and the N-terminal and C-terminal heptad repeat (HR) regions of gp41 [38]. Subsequently, interaction of the HR domains in a leucine zipper like fashion facilitates effector and target membranes to come in close proximity resulting in fusion [39]. Although the primary function of HIV Env glycoprotein is to facilitate viral entry, its role in bystander apoptosis is becoming increasingly evident [40,41].

### 2.2. Mechanism of HIV Env-Mediated Apoptosis: Role of gp120 and gp41

The gp120 subunit of HIV Env binds to CD4 and a coreceptor making it a likely candidate to mediate apoptosis. In fact, early studies showed that inhibition of gp120 binding to CD4 or the coreceptor eliminates Env mediated bystander apoptosis [42]. However, in later studies, it was observed that, while gp120 binding is required for this process, it is the gp41 subunit that mediates fusion of membranes and plays a critical role in this process [35,36,43,44]. Studies by several groups have found that membrane hemifusion between HIV-1 Env expressing cells and CD4 bystander cells correlates with Env mediated bystander apoptosis [35,36,44]. Hemifusion is a process that involves transient interaction of cellular membranes characterized by mixing of the outer leaflets of the bilayers without progression to fusion pore formation [45]. Although hemifusion has been extensively studied in influenza, it has been demonstrated by multiple independent groups in HIV as well [35,44,46,47,48]. It is hypothesized that membrane damage mediated during this hemifusion process in part mediates bystander apoptosis; a phenomenon also referred to as the “kiss of death” [31].

Further evidence for a role of HIV Env mediated fusion in CD4 loss can be found in clinical studies where presence of syncytia inducing (SI) viruses has been associated with poor prognosis and a rapid CD4 decline [49,50]. The SI phenotype is not only associated with increased pathogenesis [51] but also CXCR4 tropism [52] of viruses. In severe combined immunodeficiency humanized (SCID-hu) mouse model, SI phenotype and CXCR4 tropism is linked to CD4 loss as certain CCR5 tropic lab strains fail to induce CD4 decline [53]. The correlation of HIV mediated CD4 loss with the fusogenic activity of Env glycoprotein is also supported by animal models such as the chimeric SHIV infection in rhesus macaques [54,55,56] and HIV infection in humanized mice [26,29].

Although gp41 mediated fusion plays an important role in bystander apoptosis, the role of gp120 subunit of Env in HIV mediated bystander apoptosis cannot be ignored. As the initial contact between bystander cells and infected cells is made via the gp120 subunit, the phenotypic characteristics of gp120 determine the subsequent steps in Env mediated fusion and therefore bystander apoptosis. Increased affinity of gp120 for CD4 receptor and/or coreceptor can influence both Env mediated fusion as well as apoptosis [28,57,58,59]. We have also found a negative correlation between potential N-glycosylation sites (PNGS) and bystander apoptosis inducing phenotype among primary patient derived Envs [60]. Thus, the phenotypic characteristic of Env glycoprotein is complex and a consequence of genotypic characteristics of both gp120 and gp41 subunits.

### 2.3. HIV Env Glycoprotein Variability and Evolution

HIV Env glycoprotein is probably the most variable protein found in nature [61] and evolves throughout the course of infection within individuals [62]. One of the interesting phenomenon in HIV Env evolution is switching of coreceptor usage from CCR5 tropic (R5) early viruses to CXCR4 tropic (X4) viruses during late stages of the disease [49,50,51]. This phenotypic change in virus often precedes a rapid decline in CD4 cells suggesting an increased pathogenic potential of X4 viruses [51,63,64]. However, coreceptor switching is not a requirement for HIV mediated bystander apoptosis and it has been shown that both X4 and R5 tropic Envs are capable of inducing apoptosis in bystander cells given the appropriate coreceptor is expressed [43,65,66]. Furthermore, late stage AIDS associated R5 tropic viruses have been shown to be more pathogenic with increased replication and cytopathic effect [67,68]. These AIDS associated R5 viruses have been shown to be more fusogenic [69] and this phenotypic difference has been mapped to the glycosylation site at position Asn 362 [58]. We have found that in vitro adaptation of HIV to low levels of CCR5 results in evolution of virus to higher CCR5 affinity and increased bystander apoptosis in cell expressing low CCR5 levels [66]. An in vitro analysis of primary CCR5 tropic Envs for bystander apoptosis induction also shows that patient derived Envs vary considerably with respect to apoptosis phenotype and several genetic signatures correlated with apoptosis, including low levels of PNGS [60]. Recently, we have found that the apoptosis inducing potential (AIP) of primary Envs from patients correlates with CD4 decline and CD4:CD8 ratio in patients [10]. These studies provide strong evidence for a role of HIV Env in bystander apoptosis as well as disease progression.

### 2.4. Signaling via HIV Env Glycoprotein

The apoptotic signaling mediated by HIV Env has been studied extensively and activation of classical apoptosis markers such as caspase-3, mitochondrial depolarization [70,71] and reactive oxygen species (ROS) production [36] has been observed during the process. However, a role of apoptosis signaling ligands such as Fas and tumor necrosis factor (TNF) has not been found in Env mediated apoptosis, suggesting that it constitutes a unique signaling pathway [36,72,73]. As the gp120 subunit of HIV Env binds to CD4 and CXCR4/CCR5, signaling via either of these receptors could be important for Env mediated apoptosis. However, eliminating CD4 signaling via cytoplasmic tail truncation and CXCR4 signaling via G protein inhibitors fails to inhibit Env mediated apoptosis [34,35] suggesting that signaling via either of these receptors is not required for this phenomenon. The inhibition of HIV Env mediated apoptosis by gp41 inhibitors such as T20 (enfuvirtide) and C34 peptide indicates that the signaling may be initiated by gp41 catalyzed events such as hemifusion of membranes [21,35,74]. Moreover, although the activation of caspase-3 and mitochondrial depolarization has been seen in HIV Env mediated apoptosis [35,36,74], the specific target that initiates this process, perhaps in the plasma membrane remains undetermined.

Current evidence largely suggests that HIV-1 Env mediated apoptosis in bystander cells involves the mitochondria and is associated with ROS production [75], a global increase in cell metabolism and increase in mitochondrial fission. ROS generation mediated by Env can be inhibited by the CXCR4 antagonist AMD-3100 although it is not clear if ROS generation is a consequence of signaling upon Env binding to CXCR4 or as a result of Env mediated oxidative stress. Similarly, HIV Env is known to trigger upregulation of the stress protein family of chaperones such as Heat shock protein 70 (Hsp70). Both mitochondrial and cytoplasmic forms of Hsp70 are upregulated after contact with Env which is also upregulated in HIV infected cells functioning as an innate immunity factor [76]. Molina et al. [77] showed that this defensive response is initiated in uninfected cells after Env–CXCR4 interaction. Although heat shock proteins can both inhibit and enhance apoptosis via different pathways, reduction in cellular expression of HSp60 increases Env mediated bystander apoptosis [70]. While other proteins including the proteasome- and ubiquitin-related proteins have been known to be involved in the process, further investigation is required to delineate the precise pathway involved in HIV Env mediated bystander cell death, especially in physiologically relevant CD4 T cells.

### 2.5. Targeting HIV gp41 Can Alter Bystander Apoptosis

The inhibition of HIV Env mediated bystander apoptosis by gp41 fusion inhibitors opens the door for targeting gp41 not only to inhibit HIV infection but also bystander apoptosis [78]. In this context, it has been reported that Enfuvirtide therapy may have beneficial effects in patients by inhibiting gp41 mediated cell death [79]. However, similar to other anti-retrovirals, Enfuvirtide come with the caveat of resistance development. Interestingly, resistance against enfuvirtide in many cases comes at the expense of alteration in the Env fusogenic properties [80]. Thus, theoretically, methods that alter HIV gp41 mediated fusion could have therapeutic benefits via reducing bystander apoptosis. In support of this hypothesis, a clinical study by Aquaro et al. [81] found that certain resistant viruses emerging during enfuvirtide therapy were associated with CD4 increase in patients even after virological failure. These mutations were localized in the gp41 HR1 region that is known to regulate gp41 fusion activity [80]. Subsequently, Melby et al. [82] reported that mutations at position V38 in gp41 are associated with increase in CD4 recovery in enfuvirtide treated patients after virological failure. Further evidence for a role of gp41 in this process was shown by Cunyat et al. who found that the presence of V38A in combination with N140I mutation in gp41 was associated with reduced HIV associated cytopathic phenotype [48]. In vitro mutagenesis studies by our lab later confirmed that mutations at the V38 position, especially V38E, reduces bystander apoptosis activity in vitro [83]. The non-pathogenic nature of gp41 mutant V38E was not limited to in vitro studies but was also observed in humanized mice, where infection with V38E mutant resulted in slower CD4 decline accompanied with a lack of bystander apoptosis compared to infection with wild type virus [26]. Collectively, these findings support a strategy for targeting HIV gp41 to limit bystander apoptosis.

### 2.6. Fas, TNF/TRAIL and Other Viral Proteins in Bystander Apoptosis

Although a role of tumor necrosis factor receptor 1 (TNFR1) and Fas (CD95) pathways has not been found in Env mediated apoptosis, there is evidence that HIV infected cells show greater susceptibility to Fas induced apoptosis. Both membrane bound and soluble Fas are upregulated in HIV infected patients and correlate with disease progression [84]. Macrophages are believed to play a major role in cell death via this pathway as TNF expressed on the surface of activated macrophages can induce apoptosis in bystander T cells via TRAIL (TNF Related Apoptosis Inducing Ligand)–DR5 (Death Receptor) and Fas–Fas ligand interactions in a major histocompatibility complex (MHC) unrestricted manner [85,86]. Further impairment in cell viability has also been observed due to other viral proteins such as Nef and Vpr that mimic the biological effects of TNF, while gp120 and Vpu can exacerbate the pro apoptotic effects of TNF [87]. It has been shown that CD4 cross linking via gp120 activates the CD95/CD95L pathway [88] and Nef expressing T cells upregulate CD95L that mediates apoptosis in CD95 expressing bystander T cells [89]. Furthermore, Tat protein secreted from infected cells is capable of upregulating CD95L expression in uninfected cells, enhancing susceptibility to apoptosis via this pathway [90]. Another member of the TNF family that has been implicated in HIV induced bystander apoptosis in vivo is TRAIL/Apo2 Ligand (APO2L) as T cells from HIV^+^ patients are more prone to TRAIL mediated cell death [91]. Finally, Tat protein can induce upregulation of TRAIL in macrophages that can lead to apoptosis in bystander T cells [92].

### 2.7. Effects of HIV Env on Cell Types Other Than CD4 T Cells

While HIV is a disease affecting CD4 T cells, many other cell types also suffer the consequences of T helper cell dysfunction either directly or indirectly. These include cells of the neuronal lineage, thymocytes, CD34+ stem cells, B cells and cells of the monocytic lineage. With regards to CD34+ stem cells, both stimulatory and inhibitory effects of HIV Env on uninfected CD34+ progenitor cells have been observed. The stimulatory effects manifest as increased myeloid colony formation via indirect effects of gp160 through production of cytokines such as granulocyte monocyte-colony stimulating Factor (GM–CSF) [93]. On the other hand, the HIV gp120 has been shown to inhibit hematological colony formation via TNF-α section (from mononuclear cells) which is a potent hematopoiesis inhibitor [94]. Interestingly, HIV is also shown to induce cytopathic effects in thymocytes via induction of bystander apoptosis [95] along with aberrant positive and negative selection [96]. With regards to B cells, other than affecting all the functions that require CD4 help, HIV Env is known to stimulate B cells to differentiate into antibody secreting cells [97]. Similarly, gp120 binding to CD4 leads to reduced expression of co-stimulatory molecules such as B7 in macrophages derived from HIV infected individuals [98,99]. Finally, the effects of HIV infection on the nervous system cannot be overstated with the prevalence of HIV associated neurocognitive disorders (HAND) on the rise with increasing life span of HIV patients with access to HAART. Primarily, the effects are the result of viral proteins such as gp120 that are shed form HIV infected microglia resulting in neuronal apoptosis via caspase 3 activation, release of inflammatory cytokines and increase in permeability of the blood–brain barrier [100].

## 3. Host Factors

### 3.1. CCR5: Role of CCR5 in HIV Disease

The discovery of CCR5 and CXCR4 as co-receptors for HIV infection was a major breakthrough in HIV research [101]. While HIV variants can use CXCR4 for viral entry, most early viral isolates have been found to be R5 tropic [102,103]. This is most likely due to their better transmission potential and abundance of CCR5 expressing cells in the mucosal tissues, the natural sites for establishment of HIV infection [102,103,104,105]. The predominance of CCR5 as the major coreceptor for HIV has led to the development of multiple CCR5 antagonists as drug candidates [106]. The therapeutic potential of CCR5 antagonist Maraviroc in suppressing virus replication and virus-related pathologies in patients harboring CCR5 tropic viruses has been well documented [107]. Furthermore, the natural variations in CCR5 expression in humans, as a result of gene and promoter polymorphisms, have been linked to HIV disease progression [108,109].

### 3.2. CCR5 Polymorphisms

The role of CCR5 polymorphisms in HIV disease was first realized with the observation that patients with the CCR5Δ32 heterozygous genotype, while susceptible to infection, progress slower to AIDS than wild type (WT) genotype [110,111]. While CCR5Δ32 homozygous individuals are resistant to CCR5 tropic virus infection due to lack of functional CCR5 on the cell surface [112,113], the slower disease progression in the CCR5Δ32 heterozygous patients [111] was attributed to lower CCR5 levels on the cell surface [114]. Other factors affecting cell surface CCR5 levels in the host are several single nucleotide polymorphisms (SNPs) in the CCR5 promoter region [108,115]. Seven major SNPs in the CCR5 promoter make up eight different promoter haplotypes (HHA–HHG) and haplotypes such as HHC have been associated with slower disease progression [109,116]. Recent evidence suggests that CCR5 expression is further influenced by epigenetic factors along with activation status of T cell cells playing a role in this process [117]. While CCR5 levels have clearly been documented to play a role in HIV pathogenesis, the mechanism behind the phenomenon is only recently becoming clear.

### 3.3. CCR5 Env Interaction in HIV-Mediated Apoptosis

Two main processes via which CCR5 levels may regulate disease progression is by modulating virus infection and bystander apoptosis [65,118,119]. Virus replication and fusion mediated by different R5 tropic HIV isolates was shown to be dependent on CCR5 levels in HeLa cells expressing different levels of CCR5 [118]. Moreover, in CD4 T cell lines expressing varying levels of CCR5, bystander apoptosis mediated by the Env glycoprotein was dependent on CCR5 expression and the fusion capacity of the viral Env [65]. Interestingly, in the SCID-hu model of HIV pathogenesis, reconstitution of mice with thymic grafts from CCR5Δ32 heterozygous individuals supported virus replication without CD4 T cell depletion [67]. Our studies show that cells expressing low levels of CCR5 can support long-term HIV replication in the absence of bystander apoptosis [65,66]. Collectively, these data suggest that a different threshold of CCR5 level may be required for bystander apoptosis compared to virus replication.

Recently, Env CCR5 interactions were also found to be important for bystander apoptosis induction via a dual tropic HIV isolate, with the process being reduced by CCR5 inhibitors or mutations in the Env glycoprotein that abrogate CCR5 interaction [29]. Thus, the process of bystander apoptosis in the larger context of HIV pathogenesis seems to rely on two key aspects, the Env fusogencity and host CCR5 expression levels [29,60]. Patients harboring highly fusogenic Envs would likely be efficient in using low levels of CCR5 while patients with less fusogenic Envs would require high CCR5 levels for bystander apoptosis induction. Consistent with this idea, we have found that in the presence of low CCR5 levels in vitro, HIV evolves over time to acquire changes that help utilize low CCR5 levels accompanied by increase in bystander apoptosis [66].

### 3.4. CCR5 in Primate Models of SIV Infection

A role of CCR5 levels in SIV infections is strengthened by the observations that despite high levels of virus replication, African green monkeys (AGMs) and sooty mangabeys (SMs) do not progress to AIDS in nature. A paucity of CD4CCR5+ T cells in different tissues of five different primate species, where natural SIV infection leads to a non-pathogenic infection, indicates that CCR5 expression plays a significant role in SIV infections [120]. Furthermore, in mandrills (MNDs), lower number of CD4CCR5+ T cells in the mucosal surfaces has been linked to reduced transmission of SIVmnd from mother to infant [121]. In a recent study, Paiardini et al. [122] observed that reduced CCR5 expression in the CD4 central memory T cell compartment in SMs limits SIV infection and progression to AIDS. In the same study, CD4 T cell activation failed to upregulate CCR5 in SMs thereby protecting cells against SIV infection. Recently, an SIV such as phenotype has been reported in non-progressing HIV infected children characterized by low CCR5 expression and reduced immune activation [123].

## 4. Immune Activation

### 4.1. Immune Activation in HIV Disease

Chronic immune activation is a distinctive hallmark of pathogenic HIV infections [124,125,126]. Furthermore, activation of both CD4 and CD8 T cells as determined by expression of activation markers such as Ki67, HLA-DR, CD25 and CD38 [127,128] has been associated with HIV disease progression. Some studies have found immune activation to be a better predictor of disease progression than plasma viremia [13,14,15,129]. Furthermore, pathogenic HIV and SIV infections can be differentiated from non-pathogenic SIV infections in natural hosts by lack of the immune activation in the latter [4,126,130,131]. Correlation between immune activation and CD4 decline has also been reported in humanized mouse models of HIV infection [132]. The mechanism of immune activation in pathogenic HIV infection involves several factors including virus replication, loss of Th17 cells, disruption of intestinal barrier, leakage of gut microbes in peripheral circulation and stimulation of toll-like receptor (TLR) pathways [130,133,134].

### 4.2. Viremia and Immune Activation

The mechanism of immune activation in HIV infection remains controversial. There is strong evidence for the involvement of viremia in immune activation as both CD4 and CD8 T cell activation is higher in viremic patients [10,135,136,137]. A reduction in immune activation after initiation of HAART therapy [138,139,140,141] and association with residual viremia in patients unable to suppress virus replication [142] is also supportive of a role of viremia in immune activation. Further evidence can be found in in vitro infection of lymph node histocultures with HIV-1 that results in activation of CD4 and CD8 T cells characterized by upregulation of CD25 and HLA-DR [143]. An involvement of plasmacytoid dendritic cells in detecting virus via TLRs and mediating immune activation via Interferon-1 (IFN-1) secretion has recently been demonstrated [144,145]. Furthermore, a recent study by Cheng et al. showed that IFN-1 is involved in CD4 loss via apoptosis in humanized mouse model of HIV infection [146].

### 4.3. Microbial Translocation, Th17 Depletion and Immune Activation

The mechanism of immune activation in HIV disease remains debated and microbial translocation has been proposed [147,148,149]. Brenchley et al. demonstrated that increased levels of lipopolysaccharide (LPS) [150] and bacterial DNA [151] in HIV infected individuals correlates with immune activation. The correlation of microbial translocation with HIV disease has been reported by several independent groups [152,153,154,155]. A specific loss of CD4 T cells in the gastrointestinal tract is a hallmark of primary HIV-1/SIV infection [156,157,158,159] and likely responsible for the impaired mucosal immunity and microbial translocation [160]. Recent studies suggest that a specific loss of gut associated Th17 subset of CD4 T cells may be behind this phenomenon [161].

Th17 are Interleukin (IL)-17 producing subset of CD4 T helper cells found on mucosal surfaces that maintain intestinal barrier integrity [162]. Both HIV infection and pathogenic SIV infection in rhesus macaques are characterized by a loss of Th17 cells in the gut while in natural SIV infections this subset is preserved [161,163,164] As Th17 cells are involved in microbial clearance in the gut, a depletion of Th17 cells is linked directly to microbial translocation and immune activation in HIV infections [165]. This subset of CD4 T cells are highly susceptible to HIV infection [122] due to the expression of CCR5 at least in the gut-associated lymphoid tissue (GALT) [166]. While the mechanism of Th17 cell depletion has not been studied directly, the susceptibility of these cells to HIV infection suggests that these cells may also be susceptible to HIV mediated apoptosis. In fact, initiation of HAART at early stages in HIV infection can preserve Th17 cell function and reverse HIV associated immune activation [141]. Furthermore, strategies aimed at restoring this population have shown clinical benefits in animal models [167].

### 4.4. Toll-Like Receptors in Immune Activation

The innate immune system recognizes pathogens via a family of TLRs that modulate adaptive immune response especially to chronic infections such as HIV [168,169]. Recognition of pathogens by TLRs results in production of inflammatory cytokines making this pathway important for immune activation [170]. The correlation between immune activation and viremia suggests that TLR family of innate sensors, specifically TLR7 that senses viral RNA and TLR9 that senses unmethylated CpG viral DNA may be involved in HIV mediated immune activation [145,171]. The sensing of viral nucleic acids by TLR7 and TLR9 in plasmacytoid dendritic cells and subsequent IFN-1 production [144] has been proposed as the mechanism behind this phenomenon. O’Brien et al. have recently demonstrated that interaction of HIV Env with CD4 on plasmacytoid dendritic cells is key to dendritic cell stimulation and IFN production [172]. Furthermore, in vitro stimulation of PBMCs with TLR ligands mediates activation in CD4 and CD8 T cells [173]. Besides recognizing viral CpG DNA, TLR9 is also stimulated by CpG DNA from bacteria. Incidentally, translocation of gut microbes and plasma levels of bacterial DNA are both increased in HIV patients and correlate with immune activation [150,151]. A role of TLRs in HIV induced immune activation is also supported by the reduction in immune activation by TLR7 and TLR9 signaling inhibitors such as chloroquine and hydroxychloroquine [174,175]. How immune activation ties into CD4 apoptosis in HIV infection is an area of active research and recent studies are uncovering the relationship between these two phenomena.

### 4.5. Immune Activation and Apoptosis

Immune activation per se can induce apoptosis in cells via the process of activation-induced cell death (AICD) and has been proposed as a mechanism of CD4 loss in HIV infections [176]. However, recent studies are less supportive of this pathway for two reasons. Firstly, high levels of immune activation is also seen in CD8 T cells in HIV infected patients but the loss is largely limited to CD4 cells [10]. Secondly, experimental induction of immune activation in vivo in rhesus macaques using LPS [177] and in humanized mice [178] fails to cause a specific loss of CD4 T cells or alter the CD4:CD8 ratio. At the same time, induction of immune activation in natural SIV infection in AGMs can lead to partial CD4 loss in an otherwise non-pathogenic infection [179]. Recent studies by our group have found that activation of PBMCs in vitro enhances the susceptibility of CD4 T cells to HIV-1 Env mediated bystander apoptosis and alters CD4:CD8 ratio similar to that observed in HIV infections [10]. These findings support the hypothesis that while bystander apoptosis is largely mediated by HIV-1 Env, this process can be significantly enhanced if the cells are activated. The mechanism by which immune activation increases susceptibility of CD4 T cells to HIV Env mediated apoptosis remains to be determined. One possibility is that immune activation causes upregulation of coreceptors CXCR4 and CCR5 on cells [180,181] that not only enhances virus replication but could also potentially accelerate Env mediated apoptosis.

## 5. Other Pathways of Cell Death

### 5.1. Role of Autophagy in HIV-Mediated Cell Death

Although programmed cell death (apoptosis) is considered to be the key mechanism via which HIV causes CD4 T cells death, other cell death pathways such as autophagy [182,183] have also been proposed as mediators of T cell decline. Autophagy or type II programmed cell death is a catabolic process by which cellular cytoplasmic components and organelles are delivered to the lysosomes for degradation with the objective of establishing homeostasis after stress related stimuli [184]. Autophagy is characterized by the formation of membrane bound compartments that engulf cytoplasmic material, involves the autophagy-related (Atg) group of proteins [185] and has been described in several bacterial and viral infections including HIV [182,186,187,188]. Studies in HIV induced autophagy have found a role of the Env glycoprotein in this process via interaction with the co-receptor CXCR4 [187]. Interestingly, cells infected with HIV or those expressing the Env glycoprotein on the surface [186,187] induce autophagy in uninfected bystander CD4 T cells characterized by accumulation of Beclin 1 [187]. The process required the presence of CD4 and CXCR4 on the target cells surface, was independent of CD4 and CXCR4 signaling and could be inhibited by drugs that block autophagy such as bafilomycin A1 or siRNAs specific for *Beclin 1* and *Atg7* genes [187]. As CD4/CXCR4 signaling was not required for HIV induced autophagy, later studies identified the role of HIV gp41 in this process as fusion inhibitors (T20 and C34) or gp41 mutations (V2E) [189] inhibited Env mediated autophagy. As the mechanism of autophagy induction by HIV Env glycoprotein is similar to apoptosis, combined with the extensive cross talk between these pathways [190,191], it is plausible that apoptosis and autophagy may both play a role in CD4 T cell loss.

### 5.2. Role of Pyroptosis in HIV-Mediated Cell Death

Recent studies have suggested a role of the pro-inflammatory cell death pathway called pyroptosis [192] in HIV mediated bystander cell death. Studies by Doitsh et al. demonstrated that cell death in majority of bystander CD4 T cells is due to abortive infection of non-permissive resting CD4 T cells where there is accumulation of incomplete reverse transcription products [193,194]. These incomplete transcripts are detected by the cellular IFl16 DNA sensor to activate a pro inflammatory and pro apoptotic response characterized by activation of caspase-1 [195]. Activation of caspace-1 in quiescent T cells leads to pyroptosis, a form of programmed cell death marked by activation of caspase-1 rather than caspase-3 and release of pro-inflammatory cytokines such as IL-1 beta [196]. It has been speculated that this mechanism does not aid in clearing virus infection but rather creates a vicious cycle of inflammation by attracting new permissive cells to the site of infection. Thus, targeting caspase-1 via inhibitors such as VX-765 was suggested as a safe and viable approach to reduce HIV induced CD4 T cell death [193]. Recent studies from the same group suggest that cell to cell contact between infected and uninfected cells was essential for this form of cell death as cell free virus failed to induce pyroptosis underscoring the importance of the virological synapse in HIV pathogenesis [197]. Although pyroptosis has been suggested as an alternate pathway of cell death in HIV infection the studies are based on ex vivo human lymphoid aggregate culture model. Currently there is limited in vivo data from primate or humanized mouse model to suggest that this pathway is active in pathogenic HIV/SIV infections in vivo. In fact, a recent study by Cheng et al. failed to detect caspase-1 activation in humanized mouse model of HIV infection while apoptosis and caspase-3 activation were readily detected [146].

## 6. Model of HIV-Mediated Bystander Apoptosis

### 6.1. Detailed Model of Host and Viral Factors in HIV-Mediated Bystander Apoptosis

**Figure 1:**
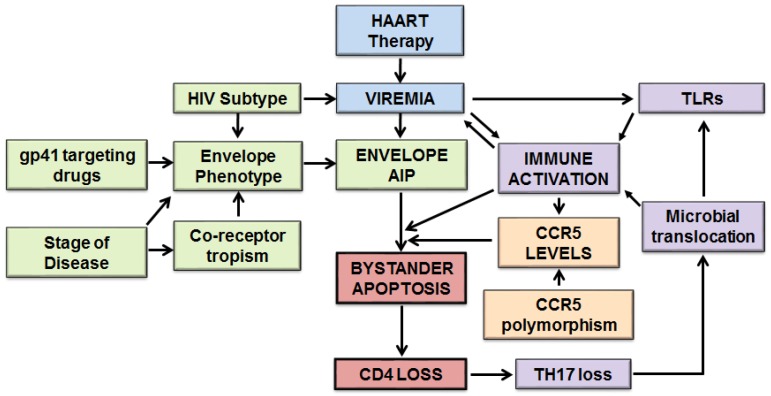
Model of host and viral factors in human immunodeficiency virus (HIV)-mediated bystander apoptosis. HIV mediated bystander apoptosis and CD4 decline can be attributed to both host and viral factors. Fundamental to this process is active virus replication (viremia) as suppressing virus replication via highly active anti-retroviral therapy (HAART) suppresses the major immunopathological variables of the disease including CD4 apoptosis and immune activation. The phenotype of the Envelope (Env) glycoprotein is another major determinant of HIV pathogenesis as the Env apoptosis inducing potential (AIP) correlates with CD4 loss. Other genotypic and phenotypic variations in Env like coreceptor usage, virus subtype, fusogenic activity of Env, etc. have been associated with disease. The binding of Env to CCR5 is also fundamental to HIV induced bystander apoptosis and variations in CCR5 levels can influence this phenomenon. Immune activation is a key immunopathological feature of HIV infections that correlates with CD4 decline and can be affected via multiple pathways as shown by arrows. TLR: toll-like receptor).

Apoptosis mediated by HIV infections is more complex than previously thought. A role of both host and viral factors in this phenomenon is becoming increasingly evident. Based on recent evidence we are proposing a detailed model of HIV mediated bystander apoptosis (Figure 1).

Fundamental to this process is active virus replication which forms the primary focus of the model. The suppression of major immunopathological variables including apoptosis with successful HAART suggests that viremia plays a key role in this process. Besides active virus replication, the phenotype of the Env glycoprotein is the other major determinant of HIV pathogenesis. Recent evidence from our lab suggests that variability in HIV Env glycoprotein in terms of apoptosis inducing potential (AIP) correlates with CD4 loss [10]. Multiple genotypic and phenotypic variations in Env have been associated with disease including coreceptor usage, virus subtype, fusogenic activity of Env, N-glycosylation sites and coreceptor binding [60,66]. The binding of Env to CCR5 is also fundamental to HIV pathogenesis and bystander apoptosis and variations in CCR5 levels as a result of CCR5 gene and promoter polymorphisms can influence this phenomenon [65,109]. Immune activation is a key immunopathological feature of HIV infections that correlates with CD4 decline. Recent evidence from our lab shows that in vitro activated PBMCs are more susceptible to HIV Env mediated bystander apoptosis [10]. These new findings help tie the role of immune activation with Env mediated apoptosis completing the complex network of major factors in HIV associated apoptosis. We also include in our model factors that influence immune activation, including a role of virus mediated TLR activation [145] as well as stimulation of TLRs via microbial translocation [150]. The translocation of microbes from the gut may be further facilitated by a specific loss of Th17 subset of CD4 T cells in the gut [161] possibly as a consequence of HIV-mediated apoptosis (Figure 1).

### 6.2. Host and Viral Factors Determining Differential CD4 Loss in HIV Infections

Based on our model above, we can speculate several factors that may influence differential CD4 apoptosis and rate of progression in HIV patients. A list of these factors is given in Table 1.

One could envision that either individually or a combination of these factors would determine different scenarios that could constitute pathogenic or non-pathogenic infections. For example, the non-pathogenic SIV infections in natural host are likely a consequence of low immune activation combined with low CCR5 expression [4,122]. In support of this scenario, experimental induction of immune activation in AGMs infected with SIV increases virus replication and CD4 T cells depletion [179]. Similarly, long term non-progressors may have low levels of CCR5 or low viremia or a combination of both [198]. A recent study in non-progressing HIV-infected children found that low levels of CCR5 combined with low immune activation is likely behind the lack of disease progression in this unique cohort [123]. In viremic non-progressors, limited immune activation [199] and limited infection of central memory cells combined with impaired viral fitness has been linked to non-progression [200]. On the other spectrum, high viremia combined with highly pathogenic phenotype of Env results is progression to AIDS as seen in several studies [10,56,201,202,203].

## 7. Conclusions

Recent findings have provided researchers with a better picture of the host and viral factors involved in HIV mediated bystander apoptosis. It is clear that this phenomenon is a complex interplay between the virus and the host. The contribution of each of these factors varies between individuals as well as viral variants. Strategies that limit immune activation, alter CCR5 levels or target Env phenotype like gp41 inhibitors remain attractive approaches to circumvent HIV pathogenesis and warrant further investigation. However, from currently available data, it is apparent that the onus of HIV mediated bystander apoptosis is largely on virus replication and phenotype of the virus. This suggests that suppressing virus replication is perhaps the best strategy to limit HIV-mediated bystander apoptosis for now.

## Figures and Tables

**Table 1:** Host and viral factors determining differential CD4 loss in HIV infections.

Factors Limiting Bystander Apoptosis	Factors Enhancing Bystander Apoptosis
➢ Poor virus replication	➢ High virus replication
➢ Low AIP phenotype	➢ High AIP phenotype
➢ Low CCR5 levels	➢ High CCR5 levels
➢ Low immune activation	➢ High immune activation
➢ Low Env CCR5 binding affinity	➢ High Env CCR5 binding affinity
➢ Less fusogenic Env	➢ Highly fusogenic Env
